# En-face widefield optical coherence tomography angiography for understanding vascular networks changes in two cases of acute retinal necrosis

**DOI:** 10.1186/s12348-023-00331-8

**Published:** 2023-03-07

**Authors:** Mami Tomita, Mizuki Tagami, Norihiko Misawa, Atsushi Sakai, Yusuke Haruna, Shigeru Honda

**Affiliations:** Department of Ophthalmology and Visual Sciences, Graduate School of Medicine, Osaka Metropolitan University, 1-5-7 Asahimachi, Abeno-ku, Osaka-shi, 545-8586 Japan

**Keywords:** En-face imaging, Acute retinal necrosis, Widefield optical coherence tomography angiography

## Abstract

**Purpose:**

The present study assesses the utility of en-face widefield optical coherence tomography angiography (OCTA) imaging for evaluating the retinal vascular network during the course of treatment in acute retinal necrosis(ARN).

**Observations:**

OCTA images of two cases of acute retinal necrosis were analyzed. Case 1 was a 15-year-old male with visual crowding in his right eye who had best-corrected visual acuity of 16/20 and intraocular pressure of 25 mmHg in his right eye on initial evaluation. Case 2 was a 57-year-old male with visual crowding in his left eye who had best-corrected visual acuity of 20/20 in his left eye on initial examination and intraocular pressure of 19.3 mmHg.

In both patients, dynamic changes could be tracked by en-face ultra-widefield OCTA imaging before and up to 1 year after surgical treatment. The images showed arteriovenous anastomosis and the nonperfused area on the surface of the retina.

**Conclusions and importance:**

En-face widefield OCTA is useful for monitoring the structure of retinal vessels over time in acute retinal necrosis. Wide-angle OCTA is used to non-invasively examine retinal vascular dynamic changes in ARN. OCTA artifacts due to intraocular inflammation appeared, making interpretation difficult. These will remain as issues in the future. It seems difficult for a while to completely replace FA due to the problem of image clarity.

## Introduction

Acute retinal necrosis (ARN) is an uncommon form of uveitis that is associated with herpes virus infection. The rate of ARN among all forms of uveitis is 1.3%–1.7% [1].

Occlusive retinal vasculitis is a typical finding in ARN, and can include capillary nonperfusion and ischemia, vascular occlusions, preretinal neovascularization, microaneurysms, and telangiectasia [2]. Fundus fluorescein angiography (FA) has high sensitivity for detecting inflammation of the retinal vessels. In active vascular disease, leakage of fluorescein and staining of the blood vessel wall indicates breakdown of the inner blood–retinal barrier [3, 4]. However, FA cannot be performed frequently because of the high risk of side effects such as nausea, vomiting, and anaphylactic shock [5]. Optical coherence tomography angiography (OCTA) cannot detect leakage but can delineate changes in the vascular density of the superficial and deep capillary plexus in vasculitis.

Recently, OCT have enabled a clear diagnosis to be obtained in a non-invasive manner [6, 7]. Here we report retinal vascular change in acute retinal necrosis that occurred over 1 year, observed using the OCTA imaging system.

### Findings

#### Case 1

**Fig. 1:**
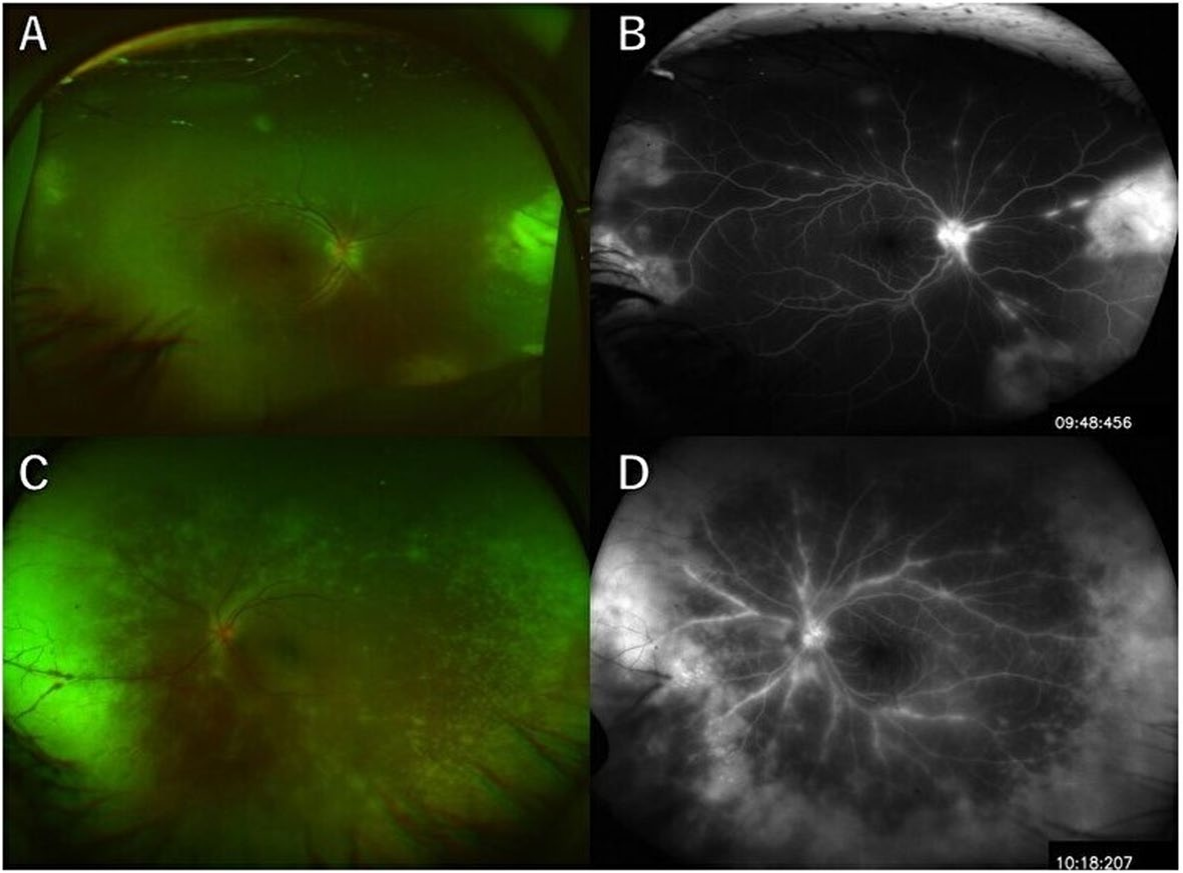
Fundus photograph shows swelling of the optic disc, yellowish-white retinal lesions with discrete borders, and retinal vasculitis in the right eye of Case 1 at the time of the first visit (**A**). Fluorescein angiography (FAG)shows leakage due to vasculitis but no nonperfusion area is seen (**B**). Fundus photograph shows swelling of the optic disc and beaded expansion of retinal blood vessels (**C**). FAG shows leakage due to vasculitis and peripheral retinal strong leakage like a frost covered tree is seen (**D**)

A 15-year-old male with visual crowding in his right eye was referred to our department for a detailed examination. Best-corrected visual acuity was 16/20 in his right eye at the initial examination, and intraocular pressure was 25 mmHg. Anterior segment slit-lamp examination revealed inflammation of the anterior segment of the eye and mutton fat keratic precipitates (KPs). Fundus examination of the right eye (Fig. 1A) revealed optic disk swelling and yellowish-white retinal lesions with discrete borders characteristic of retinal vasculitis. Fundus fluorescein angiography (FA) (Fig. 1B) revealed leakage due to vasculitis. However no nonperfusion area (NPA) was seen on FA or on the en-face widefield OCTA image (Fig. 1C). Laboratory tests were negative for human T-cell lymphotropic virus 1, syphilis, toxoplasma, and cytomegalovirus sera, and negative for the enzyme-linked immunospot assay. The patient was diagnosed with ARN and prophylactic vitrectomy with silicon oil tamponade and photocoagulation to mid-peripheral retina were done. Six months later, after confirming that there was no retinal detachment, silicone oil removal was performed. Parenteral acyclovir was started at a dosage of 10 mg/kg of body weight, three times a day for 14 days. Oral valacyclovir at 3000 mg once daily for 2 weeks was also administered. Polymerase chain reaction (PCR) analysis of a tissue sample at the department of virology detected Herpes simplex virus 1 (HSV-1) 3.55 × 10^3^ copies/mL. Approximately 1 month later, NPA was confirmed by en-face OCTA (Xephilio OCT-S1,Canon, Tokyo, Japan) (Fig. 1D, E). There was no abnormality of the retinal vessels.

The visual acuity at the final examination was 10/20, and although there was a few vitreous flare, which was thought to be due to a breakdown of the blood retinal barrier, the progression of inflammation had subsided, and retinal detachment had not appeared without the presence of tamponade substances. No improvement was observed for the retinal capillary non perfusion about 1.5 years after onset.

#### Case 2

**Fig. 2:**
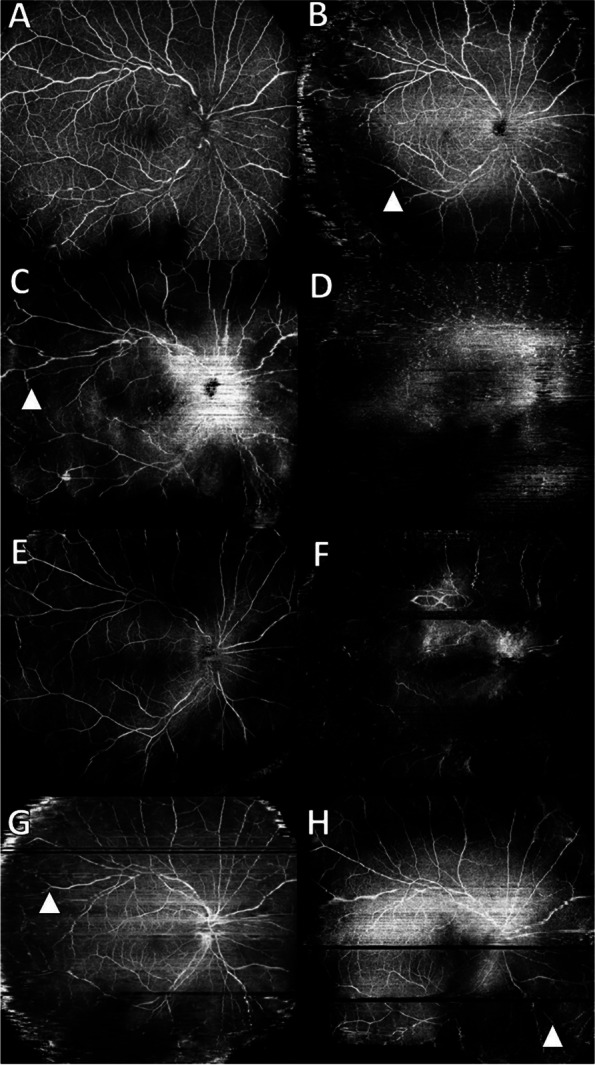
En-face ultra-widefield optical coherence tomography angiography (OCTA) images of the right eye for Case 1 (**A**-**H**). Over the course of 1 year, the peripheral avascular area expanded from the arcade vessels. **A** The first visit, **B** 1 month after surgery, **C** 2 months later, **D** 3 months later, **E** 6 months later, **E** 10 months later, **F** 1 year later, **G** After 1 year and 5 months (white arrow head: NPA) The images have several artifact

A 57-year-old male with visual crowding and conjunctival congestion in his left eye was referred to our department for a detailed examination. Best-corrected visual acuity was 20/20 in his left eye at the initial examination, and intraocular pressure was 19.3 mmHg. Anterior segment slit-lamp examination revealed mutton fat KPs in addition to anterior segment inflammation. Fundus examination (Fig. 2F) revealed optic disc swelling, yellowish-white retinal lesions and beaded expansion of retinal blood vessels. Laboratory data showed slightly positive HSV IgM. Findings were negative for other infections. FA showed beaded leakage from retinal vessels (Fig. 2G). No NPA was observed on either FA or OCTA (Fig. 2G, H). A 625 mg infusion of acyclovir (10 mg/kg bodyweight) was started three times a day and continued for 7 days, and oral valacyclovir 3000 mg was continued for 1 month. And then prophylactic vitrectomy with phacoemulsification and intraocular lens implantation (PEA + IOL) and with silicon oil tamponade was done and photocoagulation to mid-peripheral retina was done.

In this case, retinal detachment developed during surgery, it was determined that retinal detachment would likely occur if silicone oil was removed, so silicone oil was not removed.

**Fig. 3:**
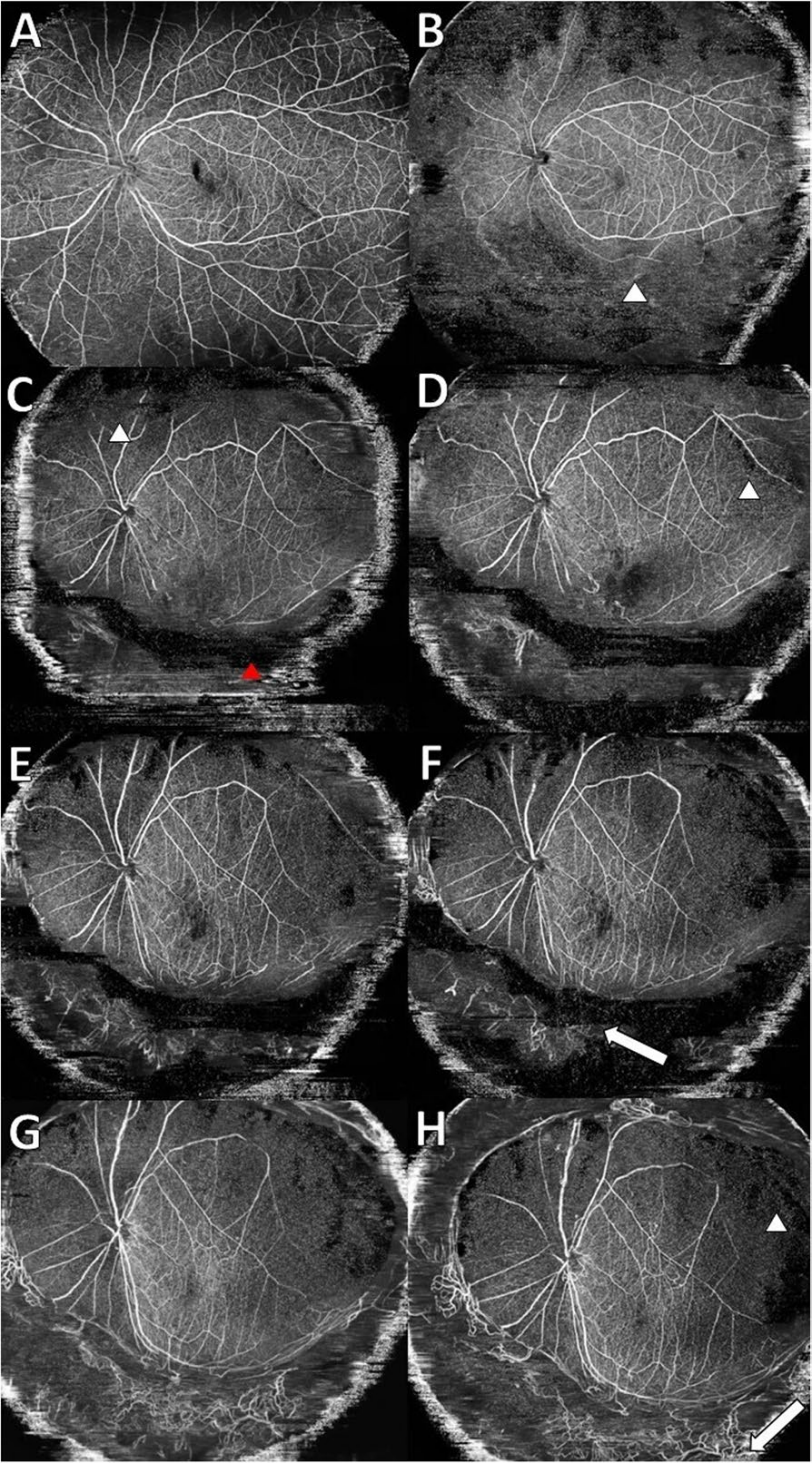
En-face ultra-widefield optical coherence tomography angiography (OCTA) images of the left eye for Case 2 (**A**-**H**). **A** The first visit, **B** 1 month after surgery, **C** 2 months later, **D** 3 months later, **E** 6 months later, **F** 10 months later, **G** 1 year later, **H** After 1 year and 4 months (red arrow head: posterior vitreous detachment line, white arrow: anastomosis) Over the course of 1 year, There is peripheral neovascularization with straightening of the vessels towards inferiorly, there seems to be traction on the macula also. The images have several artifacts

PCR analysis of a vitreous sample revealed varicella-zoster virus 6.02 × 10^6^ copies/mL. Three months after onset, numerous equatorial-to-peripheral retinal anastomotic short circuits and extensive NPA were observed on OCTA (Fig. 2I, J). The visual acuity at the final examination was 4/20, and the progression of inflammation had subsided, and retinal detachment had not appeared under silicon oil tamponade. No improvement was observed for the retinal capillary non perfusion about 1.5 years after onset (Fig. 3).

**Fig. 4:**
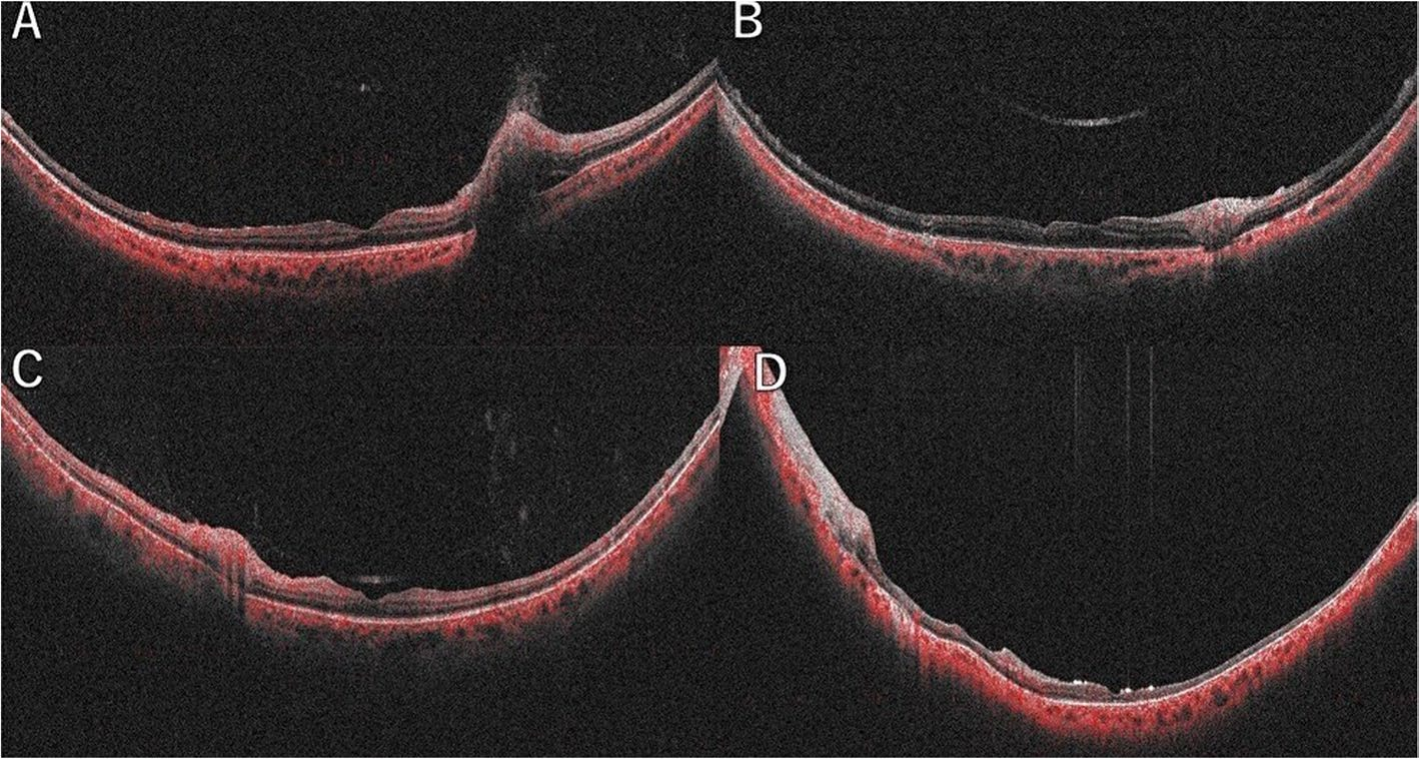
Optical coherence tomography (OCT) images of the right eye for Case 1. **A** The first visit, **B** After the treatment and the leftt eye for Case 2. **C** The first visit, D.After the treatment. In both cases, atrophy and thinning of all retinal layers are observed

In both Case 1 and 2, OCT-B scans revealed thinning of the entire sensory retina due to ARN, inflammation, and disruption of the blood–retinal barrier (Fig. 4).

## Discussion

We performed prophylactic vitrectomy in 2 cases of ARN, and observed the retinal vascular network with en-face wide filed OCTA over periods of about one and half years.

Regarding prophylactic vitrectomy, there are many reports that describe its effectiveness and rather negative ones, including prophylactic laser treatment [8–11].

No complete consensus has yet been reached at present.

In our two cases, there were no recurrence of postoperative retinal detachment and the course were uneventful.

OCT and OCTA are particularly effective for evaluating areas of telangiectasia, increased central foveal avascular zone, telangiectasia, shunts, neovascularization, and other vascular abnormalities [6, 12–14].

OCTA can demonstrate vascular remodeling due to ischemia as well as vascular revascularization after treatment in areas affected by retinal vasculitis obliterans [3, 15, 16].

Evaluation of uveitis using OCTA showed that paracentral capillary density in the shallow retinal plexus was significantly lower in eyes with retinal vasculitis compared to healthy eyes [17].

These findings suggest that OCTA could be used for quantitative assessment of the effects of intraocular inflammation.

In recently, FA was commonly used to evaluate changes in retinal vascularity and numerous studies have reported change in retinal vascularity in uveitis using OCTA, however there are few reports regarding ARN [18–22].

de Andrade et al. reported that OCTA density maps obtained during the course of treatment in patients with ARN revealed a decrease in vascular density compared with baseline and a restoration of the normal pattern at 30 days of treatment [23]. As occlusive retinal vasculitis carries a high risk of neovascularization, with a reported hazard ratio of 10.0 [13], evaluation of the fundus is important in patients with ARN. de Andrade case had an onset in ZONE3 (peripheral retina), however our two cases presented by us had an onset in ZONE1 (posterior) in which leakage from the optic nerve head was observed in FA, so the inflammation was more direct. It is thought that this is due to damage to the neural retina and capillary during this period. de Andrade’s OCTA was only an observation of the posterior pole, and we speculate that vascular inflammation reversibly recovered as the inflammation subsided.

In addition to CMV infection, other herpes viruses can infect the vascular endothelium and cause vascular obstruction and vasculitis, and previous studies have reported systemic vascular and retinal vascular occlusion, also known as retinal vascular occlusion and anastomosis [24–26].

In our 2016 study, we reported retinal vascular occlusion and arteriovenous anastomosis in a patient with neonatal CMV infection, which improved markedly after treatment with ganciclovir [24].

Based on those previous reports, we predicted acute and chronic vascular-related problems in ARN. We have used Widefield OCTA to visualize retinal vascular shunts, and consider that OCTA might help elucidate pathogenesis in ARN. Wide angle OCTA could have possibility supported to the FA method for evaluation of vaso-occlusive pathogenesis and vasculitis in viral uveitis, including ARN.

Clinically, the use of en-face widefield OCTA will enable noninvasive observation of the retinal vessels in ARN and to observe change and the status of the retinal vascular networks in two cases of ARN during the time course of one and half years.

As the number of cases increases, it will be possible to determine which layers are affected, whether the damage is irreversible, and whether damage correlates primarily with the prognosis of visual function.

There are several limitations to this study. We examined only two cases and a greater number of cases must be accumulated and evaluated. In addition, long-term follow-up has not yet been conducted because only 1 year has passed since initiation of the study. In addition, figures of OCTA have several artifacts, the images could be difficult to understand for reader. The problem with this case series is the OCTA artifact problem. Recent previous reports have pointed out these problems, however no very good idea has been found. This time, especially in case 1, OCTA artifacts due to intraocular inflammation appeared, making interpretation difficult. These will remain as issues in the future. It seems difficult for a while to completely replace FA due to the problem of image clarity.

## Conclusions

In conclusion, it is clinically very problematic that artifacts tend to appear, however we speculated that en-face wide-filed OCTA could be useful, which can continuously and comprehensively evaluate from the posterior pole to the peripheral retinal situation, particularly in the presence of retinal vascular shunt to retinal ischemia in ARN.

## Data Availability

All data generated or analyzed during this study are included in this article. Further enquiries can be directed to the corresponding author.
